# Myocardial repair of bioengineered cardiac patches with decellularized placental scaffold and human-induced pluripotent stem cells in a rat model of myocardial infarction

**DOI:** 10.1186/s13287-020-02066-y

**Published:** 2021-01-07

**Authors:** Yu Jiang, Si-Jia Sun, Zhe Zhen, Rui Wei, Nannan Zhang, Song-Yan Liao, Hung-Fat Tse

**Affiliations:** 1 Cardiology Division, Department of Medicine, Queen Mary Hospital, the University of Hong Kong, Hong Kong, SAR China; 2 Shenzhen Institutes of Research and Innovation, the University of Hong Kong, Shenzhen, China; 3 Department of Medicine, Shenzhen Hong Kong University Hospital, Shenzhen, China; 4 Hong Kong-Guangdong Joint Laboratory on Stem Cell and Regenerative Medicine, the University of Hong Kong, Hong Kong, SAR China

**Keywords:** Bioengineering cardiac patch, Decellularized placenta, Induced pluripotent stem cells, Myocardial repair

## Abstract

**Background:**

The creation of a bioengineered cardiac patch (BCP) is a potential novel strategy for myocardial repair. Nevertheless, the ideal scaffold for BCP is unknown.

**Objective:**

We investigated whether the decellularized placenta (DP) could serve as natural scaffold material to create a BCP for myocardial repair.

**Methods and results:**

A BCP was created by seeding human-induced pluripotent stem cell-derived cardiomyocytes (hiPSC-CMs; 1 × 106/cm2) onto DP. The functional and electrophysiological properties of the BCP were first characterized by in vitro analysis and optical mapping. Next, in vivo therapeutic efficacy of the BCP was evaluated in a rat model of myocardial infarction (MI), created by left descending coronary artery ligation (MI + BCP group), and compared with MI alone (MI group), transplantation of DP (MI + DP group), and hiPSC-CMs (MI + CM group). Cytokine profiling demonstrated that the BCP contained multiple growth and angiogenic factors, including vascular endothelial growth factor, platelet-derived growth factor, insulin-like growth factor-1, basic fibroblast growth factor, angiogenin, and angiopoietin-2. In vitro optical mapping showed that the BCP exhibited organized mechanical contraction and synchronized electrical propagation. RNA sequencing showed that DP enhanced the maturation of hiPSC-CMs compared with the monolayer of cultured hiPSC-CMs. At 4 weeks follow-up, the BCP significantly improved left ventricular (LV) function, as determined by LV ejection fraction, fractional shortening, + dP/dt_max_, and end-systolic pressure-volume relationship, compared with the MI, MI + DP, and MI + CM groups. Moreover, histological examination revealed that engraftment of the BCP at the infarct zone decreased infarct size and increased cell retention and neovascularization compared with the MI, MI + DP, and MI + CM groups.

**Conclusions:**

Our results demonstrate that a DP scaffold contains multiple growth and angiogenic factors that enhance the maturation and survival of seeded hiPSC-CMs. Transplantation of a BCP is superior to DP or hiPSC-CMs alone in reducing infarct size and improving cell retention and neovascularization, thus providing a novel therapy for myocardial repair following MI.

**Supplementary Information:**

The online version contains supplementary material available at 10.1186/s13287-020-02066-y.

## Introduction

Heart failure (HF) is one the most catastrophic complications of myocardial infarction (MI) due to the irreversible loss of cardiomyocytes with consequent cardiac structural abnormalities and myocardial dysfunction. It is associated with profound morbidity and is one of the leading causes of mortality [1]. Unfortunately, there are limited curative options for patients with severe HF due to the limited regenerative capacity of adult cardiomyocytes [2, 3]. Different stem cell-based therapies have been explored as novel therapeutic approaches to prompt cardiac repair and regeneration [4–6]. Although results are promising, their efficiency is limited by the poor survival and engraftment of transplanted cells. Among the routes of cell administration (intravenous, intro-coronary artery and intromyocardial), direct intromyocardial injection is a most effectiveness method for heart regeneration [7]. However, more than 90% of cells were lost within the first few days following transplantation, because huge percentage of cells spill out from the myocardium via direct intromyocardial injection and the rest cells are overcrowded without the sufficient blood, oxygen, and nutrition supply in the hostile milieu of the injured myocardium [8–10]. Cardiac tissue engineering is a new strategy for the route of cell administration; it can produce a bioengineered cardiac patch (BCP) with seeded cells then attached onto the epicardial surface of the myocardium to optimize cell retention and engraftment by providing transplanted cells with a microenvironment for tissue growth. Substantial efforts have been made to create a BCP by seeding pluripotent stem cell-derived cardiomyocytes onto different scaffold material [11–14]. Nevertheless the ideal scaffold material remains unclear. Recently, decellularized organ matrices have been investigated as natural scaffolds for tissue engineering, the decellularized whole-heart ECM has been applied to cardiac tissue engineering and shown to promote the maturation of CMs derived from pluripotent stem cells and to improve the heart function of MI animals, but are limited by their availability for clinical application [15–17]. A potential source of natural scaffold is placenta. It is easily available, exhibits a highly vascularized tissue structure, and is rich in extracellular matrix (ECM). After decellularization, the placenta still contains a number of growth factors and abundant ECM. Therefore, decellularized placenta (DP) is considered as an ideal natural material for tissue engineering [18–21]. In this study, we aim to investigate that decellularized placenta can be used as a natural scaffold to create a BCP with human-induced pluripotent stem cell-derived cardiomyocytes (hiPSC-CMs) for myocardial repair in a rat model of MI.

## Methods

### Ethic statement

All animal experiments conform to the guidelines from Directive 2010/63/EU of the European Parliament on the protection of animals used for scientific purposes and were approved by the Committee on the Use of Live Animals in Teaching and Research (Ref.4560-17) at the University of Hong Kong.

### Decellularization of rat placenta

**Fig. 1:**
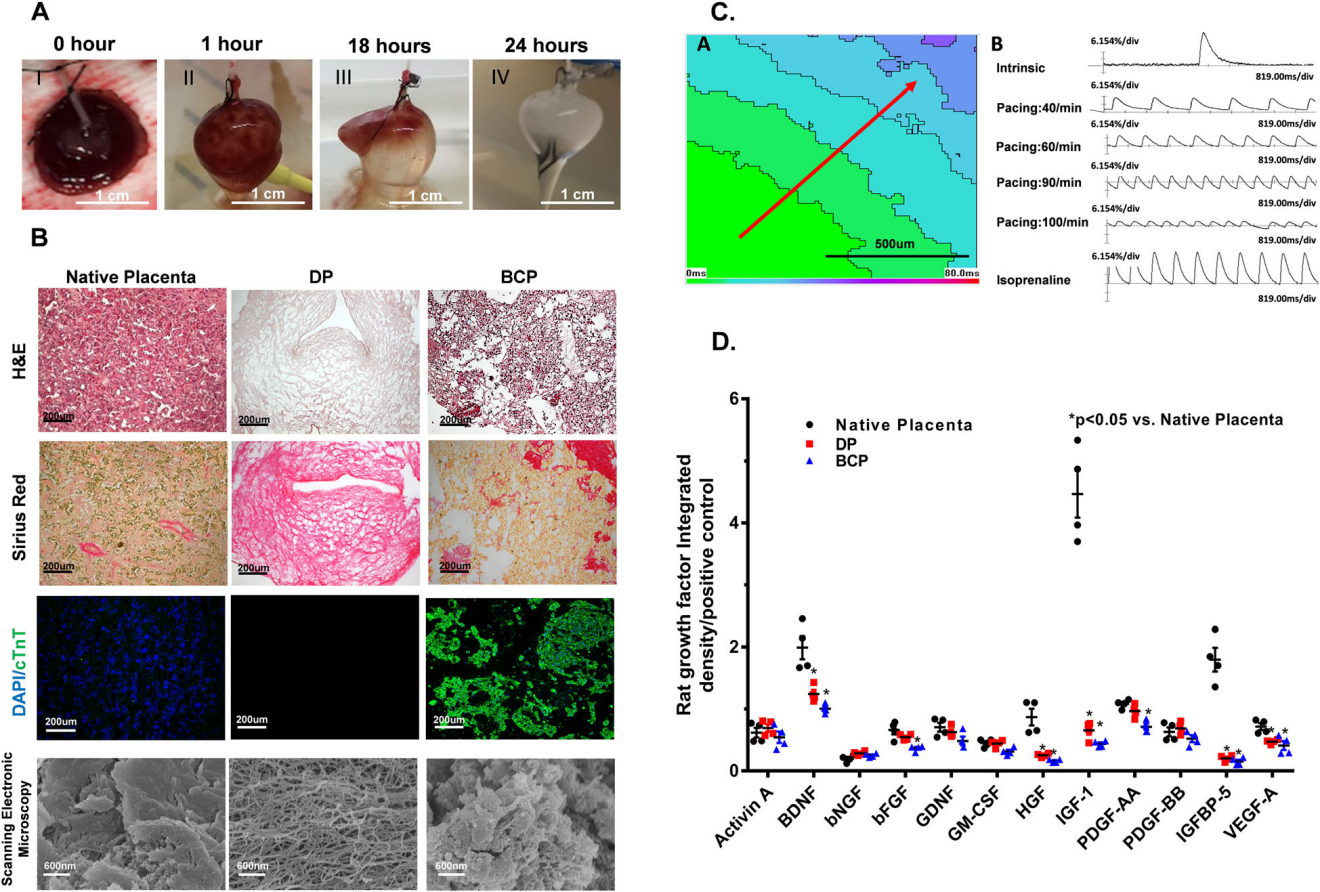
Generation and characterization of BCP. **a** Representative images of the process of decellularization of rat placenta. (I) Native placenta before decellularization; (II) after perfusion with 1%SDS for 1 h; (III) after perfusion with 1%SDS for 18 h; (IV) after perfusion with 1%SDS for 24 h. **b** Hematoxylin and eosin (H&E), Sirius Red, and DAPI/cTnT staining of sections from the native rat placenta (left), DP (Middle), and BCP (Right). The staining indicated that the cellular component and visible nuclei were removed successfully from the placenta, and DP scaffold was recellularizated with iPSC-CMs to generate a BCP. Scanning electron micrographs showed the composite of materials and network in the DP scaffold with high porosity and recellularized with hiPSC-CMs after seeding in the BCP. **c** (A) The activation map of BCP was evaluated after 7 days by staining with a Ca^2+^-sensitive dye (5 μM fura-2, AM), then measurement of the intensity of transmitted light (interval: 8 ms, sampling time:2 ms/frame). Bar, 500 μm. (B) Representative optical traces of intracellular Ca^2+^ transient at the intrinsic rate and electrical pacing at a rate of 40 times/min, 60 times/min, 90 times/min, 100 times/min, and with Isoprenaline 10 μm/ml perfusion (*x*-axis: time, *y*-axis: dF/F). **d** Quantification of multiple rat pro-survival and/or stem cell-recruiting factors (Activin A, brain-derived neurotropic factor [BDNF], beta-nerve growth factor [bNGF], glial cell-derived neurotrophic factor [GDNF], Granulocyte-macrophage colony-stimulating factor [GM-CSF] and insulin-like growth factor binding protein 5 [IGFBP-5]); and pro-angiogenic factors (basic fibroblast growth factor [bFGF], hepatocyte growth factor [HGF], insulin-like growth factor 1 [IGF-1], platelet-derived growth factor [PDGF]-AA, PDGF-BB and vascular endothelial growth factor [VEGF]) in the native rat placenta, DP and BCP. Error bars represent mean ± SEM of four independent experiments. Comparisons of native placenta, DP and BCP were performed using one-way ANOVA followed by Tukey’s post hoc test. *P < 0.05 compare with native placenta

Rat placentas were harvested from 18~20-day pregnant rats after intraperitoneal administration of ketamine (80 mg/kg) and xylazine (10 mg/kg). The rat placentas were isolated and then rinsed in phosphate-buffered saline several times. The umbilical arteries were cannulated using 25-gauge catheters and perfused with 1% sodium dodecyl sulfate (Affymetrix, Ohio, USA) at 3 ml per minute using our custom-made perfusion system at room temperature for approximately 24 h until the placentas became translucent. Then, the perfusion solution was changed to 1% Triton X-100 for a further hour to remove the residual cell components. Finally, the placentas were perfused with phosphate-buffered saline for at least 24 h to remove residual Triton X-100 until no further sign of stickiness was evident (Fig. 1a). These DP were stored at 4 °C in PBS with 1% Gibco™ Antibiotic-Antimycotic (Thermo Fisher Scientific, NY, USA).

To assess successful decellularization of the placenta, DNA quantification of the DP was performed. DNA was isolated from approximately 100 mg of native placenta and decellularized tissue using TaKaRa MiniBEST Universal Genomic DNA Extraction Kit (TaKaRa Clontech, Shiga, Japan), according to the manufacturer’s instructions, and quantified using NanoDrop 2000 (Thermo Fisher Scientific Inc., MA, USA).

### Differentiation of hiPSC-CMs

In brief, hiPSC-CMs were differentiated from a KS1 hiPSC line using a small-molecule based protocol to inhibit the Wnt/β-catinin pathway as described [22]. KS1 hiPSCs were dissociated into single cells using accutase (Invitrogen, CA, USA) and re-seeded on day 1 in a Matrigel-coated 6-well plate at a density of 5 × 10^5^ cells/well in mTeSR™ 1 medium supplemented with the ROCK inhibitor, Y27632 (5 μM) (Stemgent, MA, USA). From day 2, medium was changed daily to only mTeSR™ 1 for 3 days until confluence reached 60–90%. To increase the purity of hiPSC-CMs, and to eliminate the non-cardiomyocyte population, differentiated cells were subjected to glucose starvation according to the modified protocol [23].

The hiPSC-CMs were dissociated into single cells by accutase for 5–10 min, then collected and re-suspended in DMEM/F12 (1:1) medium supplemented with 5% fetal bovine serum (Thermo Fisher Scientific, NY, USA) to inactivate and dilute accutase. Then hiPSC-CMs were centrifuged at 200*g* for 5 min and re-suspended in the same medium for flow cytometry assessment and generation of a BCP.

### Generation and assessment of the BCP

The hiPSC-CMs were seeded on the DP scaffold at a density of 1 × 10^6^ cells /cm^2^. After 30 min, the culture medium mentioned above was added to the plate. BCP contraction was observed the day following cell seeding (Supplemental material online, video A). A 7-day cultured BCP (10 mm × 10mm) was used for transplantation.


**Additional file 6: Supplementary Video A.** BCP showed spontaneous contraction on day 7 after cell seeding.

### Histological assessment of the BCP

Hematoxylin and eosin, Picrosirius red (ab15681, Abcam, CB, UK), cTnT (ab8295, Abcam, CB, UK), and DAPI staining were used to detect the collagen component and cell nuclei before and after decellularization and scaffold recellularization. Surface and composition of the placenta, DP, and BCP were assessed using a scanning electron microscope (LEO 1530, LEO Electron Microscopy Inc., NY, USA). Samples were fixed with 4% paraformaldehyde for 24 h, then transferred to 2.5% GTA (8 h) and 1% osmium tetroxide (3 h) successively. Thereafter samples were dehydrated with series grades of ethanol (50%, 70%, 90%, and 100%). To investigate the cell maturation state of the BCP prior to transplantation, a Philips CM100 Transmission electron microscope (TEM, MA, USA) was used to compare the inner structure of hiPSC-CM aggregates and the BCP. Samples were embedded, cut into 70-nm-thick sections, and stained with uranyl acetate and lead citrate. Digital images were captured from each group from a randomly selected pool of 15 fields.

### Electrophysiological properties

Optical mapping was performed to assess the electrophysiological properties of the BCP. The BCPs (*n* = 5) were stained with 5 μM fura-2, AM (Thermo Fisher Scientific, OR, USA) in the dark for 20 min. After washing twice with Tyrode solution (NaCl 140 mM, KCl 5 mM, MgCl_2_·6H_2_O 1 mM, HEPES 5 mM with ddH_2_O and supplemented with CaCl_2_ 1.8 mM, glucose 10 mM, pH 7.3–7.4), the BCPs were incubated with Tyrode for a further 15 min to allow complete de-esterification of intracellular AM esters. Then, the BCPs were transferred to a temperature-controlled chamber containing Tyrode solution. MiCAM ULTIMA cardiac imaging system (SciMedia, CA, USA) was used to acquire the fluorescent signal through a × 10 Leica Objective Lens (Carl Zeiss, Oberkochen, Germany). Fluorescent signals that represented calcium transition of hiPSC-CMs on the BCP were collected with a resolution of 100 × 100 pixels at the 8 ms/frame, covering an area of 1 mm^2^ over 8 s. The equation of the conduction velocity of two points (*X*1, *Y*1), (*X*2, *Y*2) of BCP is √(*X*1 − *X*2) ^2 + (*Y*1 − *Y*2) ^2 /(*t*1 − *t*2). Data were analyzed by BV-Analyze software (SciMedia, CA, USA).

### Cytokine profiling

The cytokine profile of the placenta, DP, and BCP were quantified using a Rat Growth Factor Array (AAR-GF-1-2, RayBiotech, Georgia, USA) to measure the level of several pro-survival and/or stem cell-recruiting factors (activin A, brain-derived neurotropic factor, beta-nerve growth factor, glial cell-derived neurotrophic factor, granulocyte-macrophage colony-stimulating factor, and insulin-like growth factor binding protein 5) and pro-angiogenic factors (basic fibroblast growth factor, hepatocyte growth factor, insulin-like growth factor-1, platelet-derived growth factor, and vascular endothelial growth factor [VEGF]). Quantitative analysis of the membranous spots was performed with a ChemiDoc MP Imaging System (Bio-Rad, CA, USA). To control for background signals of each membrane, the concentration of each growth factor spot was calculated relative to a control spot on the same membrane.

### Paracrine functions

To assess the potential paracrine secretions from the BCP, 5 × 105 hiPSC-CMs were seeded onto DP in a Matrigel-coated plate or a Matrigel-only-coated 6-well plate. Cultures were maintained in serum-free medium at 37 °C with medium changed every other day. DP alone cultured on a Matrigel-coated plate was also included for comparison. After 1 week of culture, 2 ml of the conditioned medium was collected and analyzed for the presence of cytokines (VEGF, epidermal growth factor, hepatocyte growth factor, basic fibroblast growth factor, heparin-binding epidermal growth factor-like growth factor, platelet-derived growth factor, placental growth factor, leptin, angiogenin, and angiopoietin-2) using a human angiogenesis antibody array (QAH-ANG-1-1, RayBiotech, Georgia, USA), according to the manufacturer’s instructions.

### Illumina Nova Seq 6000 sequencing and data analysis

RNA sequencing and gene expression profiles were performed to determine the maturation of hiPSC-CMs in isolated culture versus BCP. Isolated total RNA was isolated from cultured hiPSC-CMs and BCP samples using a RNeasy Blood and Tissue kit (Qiagen, Carlsbad, CA, USA). To construct the sequencing library for Nova Seq 6000, 2 μg of total RNA was used for library construction with the Illumina TruSeq Stranded total RNA Library Prep Kit (cat # 20020596, CA, USA). Next, paired-end sequencing was performed using the Illumina Nova Seq 6000 sequencing instrument with NovaSeq 6000 S2 reagent kit (cat # 20012860, CA, USA), according to the manufacturer’s instructions, yielding 150-bp paired-end reads. EdgeR software was used for differential expression analysis of RNA sequencing and gene expression profiles with three biological replications. This analysis assumed a negative binomial distribution for statistics. To eliminate biological variation, the screening of differential genes needed to be evaluated in terms of difference multiples and significance levels. The screening threshold for differential genes in this analysis was set to |log2 (Fold Change) | > 1, FDR < 0.01.

### Quantitative real time PCR

Extracted total RNAs were reverse transcribed into complementary DNAs (cDNAs) using a High-capacity cDNA Reverse Transcription Kit (Thermo Fisher Scientific, OR, USA) according to the manufacturer’s manual. For reverse transcription of RNAs with DNA contamination, PrimeScript RT Reagent Kit with gDNA Eraser kit (TaKaRa Clontech, Shiga, Japan) was used according to the user manual. Quantitative RT-RCR was performed with the SYBR Green QPCR system (Bioscience, CA, USA) and GAPDH as an internal control (the primer list was supplied in Supplementary material online, Table S1)*.*

### Animal experiments

MI was induced in male SD rats (250–300 g) by direct left anterior descending coronary artery ligation under anesthesia with ketamine (80 mg/kg) and xylazine (10 mg/kg) as previously described [24]. Animals were randomly assigned to injection of saline (MI group, *n* = 9) or hiPSC-CMs (1 × 10^6^, MI + CM group, *n* = 9) into the myocardium, or transplanted with DP (MI + DP group, *n* = 9) or BCP (MI + BCP group, *n* = 9) at the infarcted site (Supplemental material online, Figure S1). Control animals (control group, *n* = 9) underwent open-chest surgery but without coronary ligation. All animals received immunosuppression therapy of a daily subcutaneous injection of cyclosporine A (5 mg/kg) and methylprednisolone (2 mg/kg).

### Assessment of cardiac function

Serial changes in cardiac function were measured by transthoracic echocardiogram and invasive hemodynamic assessment. Left ventricular (LV) contractile performance was examined 4 weeks after BCP transplantation by echocardiography on a high-resolution ultrasound system (Vivid-I, GE Medical Systems, WI, USA) equipped with a 10S-RS sector transducer (4.4–11.5 MHz). M-Mode images at the level of the mid-papillary muscle were obtained to measure LV end-systolic diameter (LVESD) and LV end-diastolic diameter (LVEDD) for calculation of LV ejection fraction (LVEF) and fractional shortening (FS) as described previously (Supplemental material online, Figure S2A) [25]. Invasive hemodynamic study was performed 4 weeks after transplantation before sacrifice. Animals were anesthetized and a pressure-volume conductance catheter advanced into the LV via the right carotid artery. An ADVantageTM Pressure Volume System (Scisense Inc., Ontario, Canada) was used to record LV end-systolic pressure (ESP), end-diastolic pressure (EDP), and maximal positive derivatives of LV pressure (± dP/dt_max_). The end-systolic pressure-volume relationship (ESPVR) was also measured following inferior vena cava occlusion to assess LV contractile function (Supplemental material online, Figure S2B).

### Histological assessments and immunofluorescence staining

Four weeks after BCP transplantation, animals were sacrificed, and their heart harvested for further histology study and protein analysis. Paraffin-embedded sections were stained with Masson’s trichrome to calculate infarct size, based on the average of 3 sections sampled at 2-mm intervals from the apex to the site of scar tissue in the LV free wall surface. For immunohistochemistry, human nuclei antibody (MAB1281, Millipore, Darmstadt, Germany), Anti-cTnT antibody (ab8295, Abcam, CB, UK), and anti-α-smooth muscle actin (A2547, Sigma, MO, USA) were used to detect cell survival and neovascularization respectively. The HNA-positive cells were calculated in 15 different fields from three different layers of sections using AxioVision Rel. 4.5 software (Zeiss, GmbH, Oberkochen, Germany).

### Western blotting

The infarct border zone tissue was homogenized in RIPA buffer with proteinase inhibitor for 1 h at 4 °C. Proteins were collected by centrifugation, and the supernatant boiled with 4× Laemmli sample buffer at 96 °C for 10 min. The denatured proteins were separated on a 10% SDS-PAGE gel and transferred to a membrane. The membrane was incubated with anti-VEGF (Santa Cruz, 1:1000), anti-angiogenin (Abcam, 1:1000), anti-angiopoetin 2 (Abcam, 1:1000), and b-actin (Abcam, 1: 4000) antibodies overnight at 4 °C. After washing, anti-rabbit HRP secondary antibody was added and target proteins were visualized using Clarity ECL Western Blotting Substrate (Bio-Rad, Hercules, CA, USA). Quantification of protein bands was performed using ImageJ software.

### Statistical analysis

All data are expressed as mean ± SEM. Analysis of the echocardiographic, invasive hemodynamic, and histological measurements was performed in a blinded fashion. Student’s *t* test was used to compare two groups. Comparison of variables between multiple groups was performed using one-way ANOVA with Tukey’s test. Statistical significance was defined as a *P* value < 0.05. All statistical analyses were performed using SPSS software (SPSS, Inc., Chicago, IL, USA).

## Results

### Characteristics of the BCP

As shown in Fig. 1b, hematoxylin and eosin staining confirmed that the decellularization process removed most cell components successfully and no cell debris was observed. Picrosirius red staining showed that the collagen component was retained. Moreover, both hematoxylin and eosin and DAPI staining confirmed there were no visible nuclei within the DP. After 7 days of seeding of hiPSC-CMs on the DP, immunofluorescence staining for cTnT-positive hiPSC-CMs confirmed the successful engraftment of hiPSC-CMs to create a BCP (Supplemental material online, Figure S3A). Furthermore, immunofluorescence staining for cTnT and scanning electron microscopy demonstrated repopulation of a high porosity DP scaffold with cTnT-positive hiPSC-CMs after cell seeding (Fig. 1b). As shown in Supplemental material online, Figure S3B, DNA content was dramatically depleted in the DP compared with the native placenta, and the increased DNA content of the BCP also confirmed the successful recellularization after seeding of hiPSC-CMs.

Optical mapping of the BCP was performed to determine its electrophysiological properties. As shown in the activation map (Fig. 1c), the electrical signal was conducted in a linear pattern along the BCP with a conduction velocity of 1.46 ± 0.07 cm/s (*n* = 5, Supplemental material online, video B). After administration of 10 μmol isoprenaline, the conduction velocity increased to 2.86 ± 0.04 cm/s (*n* = 5, Supplemental material online, video C). Moreover, the calcium transient and the mechanical contractility of the BCP were recorded simultaneously at a pacing rate of 40–100 beats per minute and demonstrated synchronized electromechanical propagation of the BCP according to the external electrical stimulation (Fig. 1c).


**Additional file 7: Supplementary Video B.** Intrinsic activation map and calcium transient trace on the BCP without any stimulation.


**Additional file 8: Supplementary Video C.** Activation map and calcium transient trace on the BCP under 10umol Isoprenaline perfusion.

The presence of different growth factors in native placenta, DP, and BCP were analyzed using a rat growth factor antibody array. Compared with native placenta, the level of several growth factors including brain-derived neurotropic factor, hepatocyte growth factor, insulin-like growth factor binding protein 5, insulin-like growth factor-1, platelet-derived growth factor-AA, and VEGF-A was significantly decreased in the DP (Fig. 1d, *P < 0.05*). Nevertheless, the DP and BCP had similar levels of activin A, beta-nerve growth factor, basic fibroblast growth factor, glial cell-derived neurotrophic factor granulocyte-macrophage colony-stimulating factor, and platelet-derived growth factor-BB to the native placenta (Fig. 1d, *P* > 0.05). This indicated that the DP still contained many important growth and pro-angiogenic factors that may enhance the survival and maturation of transplanted hiPSC-CMs and promote neovascularization and myocardial repair.

### BCP enhanced the paracrine effects of hiPSC-CMs

**Fig. 2:**
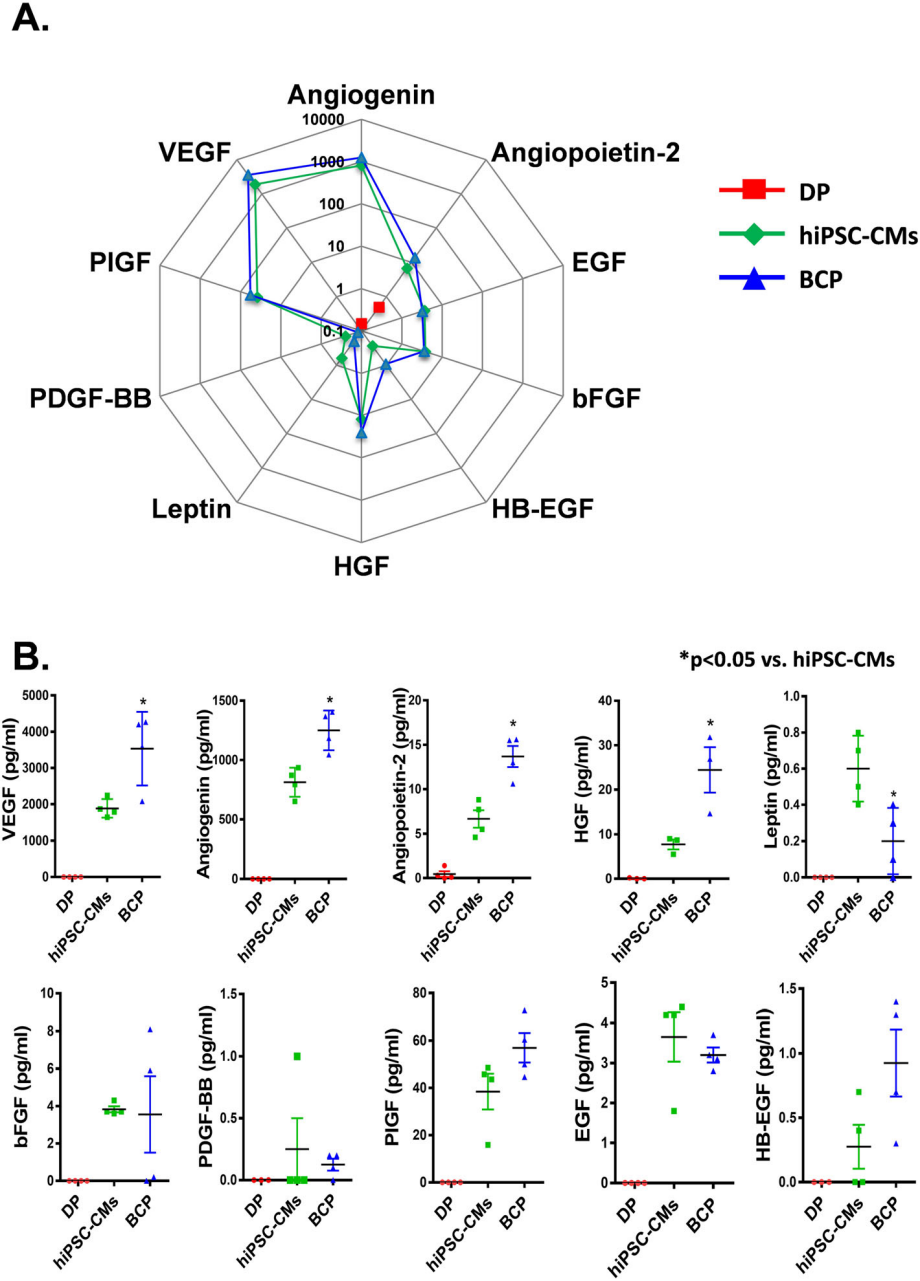
BCP improved the cell secretion of growth factors. **a** Supernatant of serum-free culture medium at day 7 was collected for comparison of the secretion of multiple human growth and angiogenesis factors in the DP, hiPSC-CM, and BCP groups. **b** Quantification of cytokines and angiogenesis factors (vascular endothelial growth factor [VEGF], epidermal growth factor [EGF], hepatocyte growth factor [HGF], basic fibroblast growth factor [bFGF], heparin-binding EGF-like growth factor [HB-EGF], platelet-derived growth factor-BB [PDGF-BB], placental growth factor [PlGF], leptin, angiogenin, and angiopoietin-2) secreted by hiPSC-CMs in the DP, CM, and BCP groups. Error bars represent mean ± SEM of Four independent experiments. Three-group comparisons were performed using one-way ANOVA followed by Tukey’s post hoc test. *P < 0.05 compared with hiPSC-CM group

To further investigate whether seeding of hiPSC-CMs on the DP scaffold could enhance the paracrine effects of hiPSC-CMs, supernatant of serum-free culture medium of the BCP at day 7 was tested using a human growth factor array. The expression of several angiogenic growth factors including VEGF, angiogenin, angiopoietin-2, and hepatocyte growth factor was significantly increased after hiPSC-CM seeding onto DP scaffold to generate a BCP, compared with monolayer cultured hiPSC-CMs on a Matrigel-coated plate (Fig. 2, *P <* 0.05).

### BCP enhances the maturation of hiPSC-CMs

**Fig. 3:**
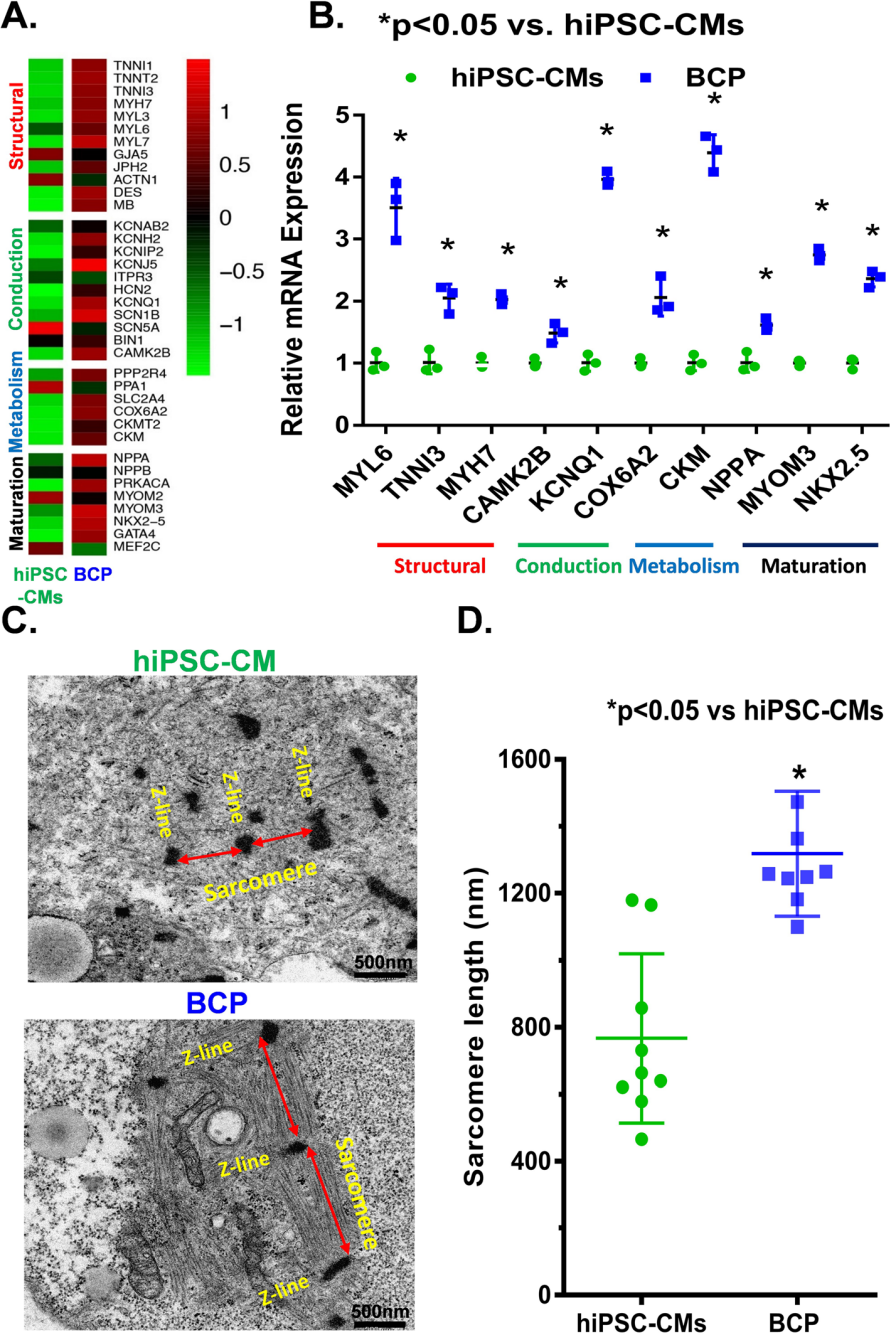
The BCP improved maturation of hiPSC-CMs. **a** Hot map of RNA sequencing, those selected representative cardiac maturation-related genes showed significant upregulation in the BCP compared with the monolayer cultured hiPSC-CMs. **b** Quantitative PCR analyses reconfirmed the upregulated expression of representative cardiac-associated genes, including cardiomyocyte structural genes (TNNI3, MYL6, and MYH7), cardiac conduction-related gene (CAMK2B, KCNQ1), cardiac metabolism-related gene (COX6A2, CKM), and cardiomyocyte maturation markers (NPPA, MYOM3, NKX2.5) as normalized to GAPDH expression. **c** Transmission electron microscope was used to evaluate the inner cell composition and ultrastructure of the BCP and the hiPSC-CM aggregates on the monolayer culture, Scale bar = 500 nm. **d** The myofibrillar sarcomere length of hiPSC-CMs on the BCP was significantly longer than that of the hiPSC-CMs. All error bars show mean ± SEM of three independent experiments. Student’s *t* test was used to compare hiPSC-CM and BCP groups. *P < 0.05 compare with hiPSC-CM group

To investigate whether the DP scaffold could improve the development and maturation of hiPSC-CMs, detailed analysis of the relative expression of selected cardiac-specific genes and key cardiac maturation genes in the BCP was compared with those of the monolayer culture of hiPSC-CMs using RNA sequencing (Supplemental material online, Figure S4). Comprehensive analysis demonstrated that the cardiomyocyte structural-related gene (TNNI1, TNNT2, TNNI3, MYH7, MYL3, MYL6, MYL7, GJA5, JPH2, ACTN1, DES, MB), cardiac conduction-related gene (KCNAB2, KCNH2, KCNIP2, KCNJ5, ITPR3, HCN2, KCNQ1, SCN1B, SCN5A, BIN1, CAMK2B), cellular metabolism-related gene (PPP2R, PPPA1, SLC2A4, COX6A2, CKMT2, CKM), and cell maturation-related gene (NPPA, NPPB, PRKACA, MYOM3, NKX2.5, GATA4) were upregulated in the BCP (Fig. 3a).

Quantitative RT-RCR further confirmed that the expression of those representative genes was significantly upregulated in the BCP compared with hiPSC-CMs (Fig. 3b, *P <* 0.05). Moreover, transmission electron microscopy revealed that the hiPSC-CMs on BCP showed more mature and organized sarcomeric structure than those on the monolayer culture (Fig. 3c). Indeed, the average sarcomere length of hiPSC-CMs on the BCP was significantly longer than that of hiPSC-CMs on the monolayer culture (Fig. 3d). Taken together, these results demonstrated that seeding of hiPSC-CMs onto the DP scaffold improved their maturation.

### BCP improves cardiac function after MI

**Fig. 4:**
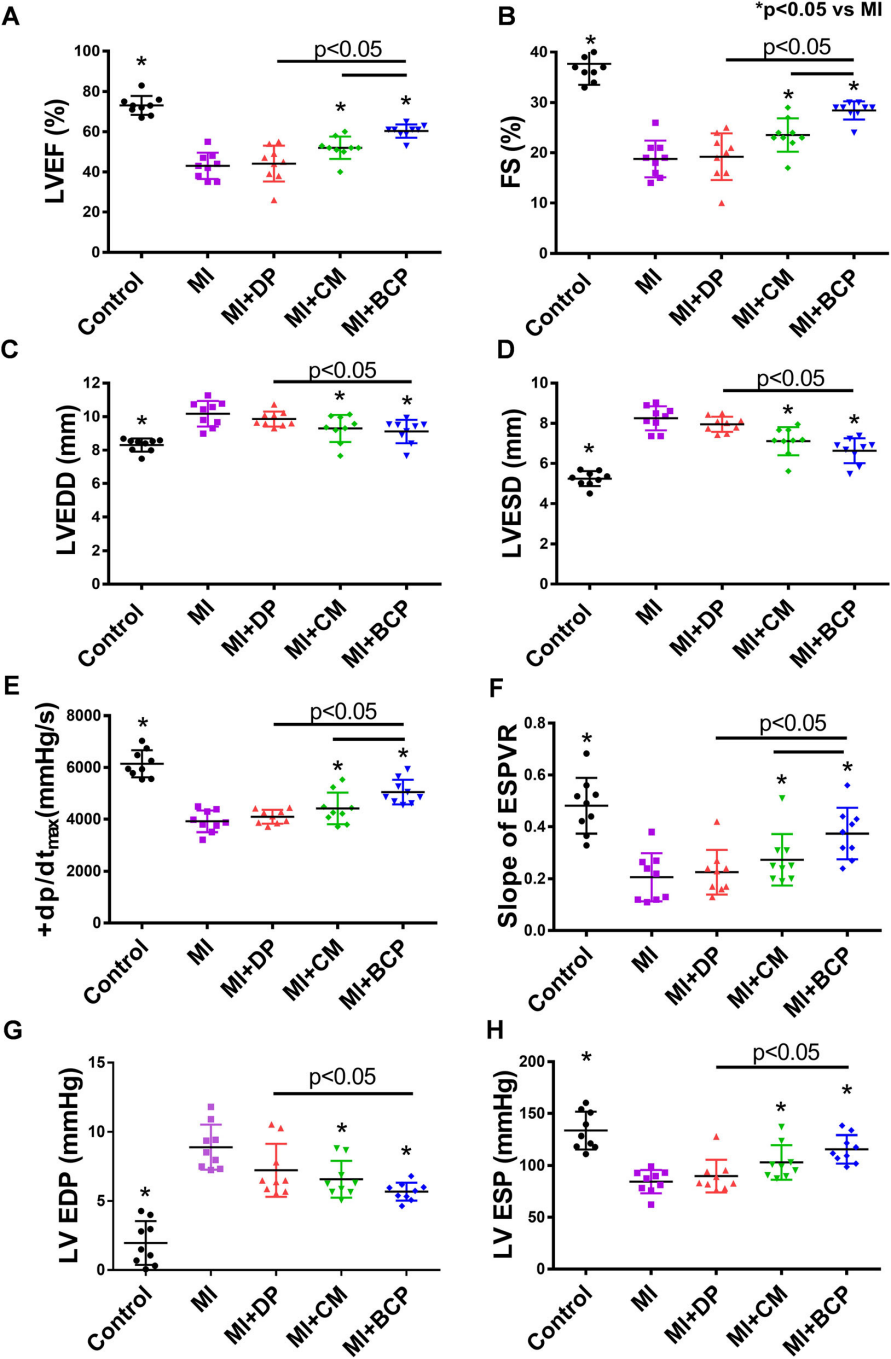
BCP improved heart function 4 weeks after transplantation. **a** Left ventricular (LV) ejection fraction (LVEF), **b** fractional shortening (FS), **c** left ventricular end-diastolic diameter (LVEDD), and **d** left ventricular end-systolic diameter (LVESD) were measured by echocardiogram to show that transplantation of hiPSC-CMs or BCP after MI significantly increased LVEF and FS, and decreased LVEDD and LVESD compared with MI group. **e** Maximal positive derivatives of LV pressure (± dP/dt_max_), **f** end-systolic pressure-volume relationship (ESPVR), **g** LV end-systolic pressure (ESP), and **h** LV end-diastolic pressure (EDP) were evaluated by invasive hemodynamic assessment to show that transplantation of hiPSC-CMs or BCP significantly increased + dP/dt_max_, ESPVR, and ESP and decreased EDP compared with the MI group. Moreover, transplantation of a BCP further increased ESPVR as compared with transplantation of DP or hiPSC-CMs alone (*P* < 0.05). Data are shown as mean ± SEM. *n* = 9, Five-group comparisons were performed using one-way ANOVA followed by Tukey’s post hoc test. *P < 0.05 compared with MI group

Echocardiographic examination was performed to assess LV function and dimensions (Supplemental material online, Figure S2A). As shown in Fig. 4a–d, LVEF and FS decreased, and LVEDD and LVESD increased in the MI group compared with control (*P <* 0.05). Implantation of DP alone after MI neither increased LVEF or FS, nor decreased LVEDD or LVESD compared with the MI group (Fig. 4a–d, *P* > 0.05). On the contrary, transplantation of hiPSC-CMs or BCP after MI significantly increased LVEF and FS, and decreased LVEDD and LVESD (Fig. 4a–d, *P <* 0.05). Moreover, transplantation of BCP further increased LVEF and FS compared with transplantation of DP or hiPSC-CMs alone after MI (Fig. 4a, b, *P <* 0.05).

Invasive hemodynamic assessment of pressure-volume loop was performed to measure LV performance (Supplemental material online, Figure S2B). As shown in Fig. 4e–h, + dP/dt_max_, ESPVR, ESP, and EDP decreased in the MI group compared with control (*P <* 0.05). Implantation of DP alone after MI neither increased + dP/dt_max_, ESPVR, and ESP nor decreased EDP compared with the MI group (Fig. 4e–h, *P* > 0.05). In contrast, transplantation of hiPSC-CMs or BCP after MI significantly increased + dP/dt_max_, ESPVR, and ESP and decreased EDP compared with the MI group (Fig. 4e–h, *P <* 0.05). In addition, transplantation of BCP further increased + dP/dt_max_ and ESPVR compared with transplantation of DP or hiPSC-CMs alone after MI (Fig. 4e–h, *P <* 0.05). Taken together, our results show that transplantation of a BCP was superior to DP or hiPSC-CMs alone for improvement of LV function in post-MI HF.

### Engraftment of a BCP reduces infarct size and promotes neovascularization after MI

**Fig. 5:**
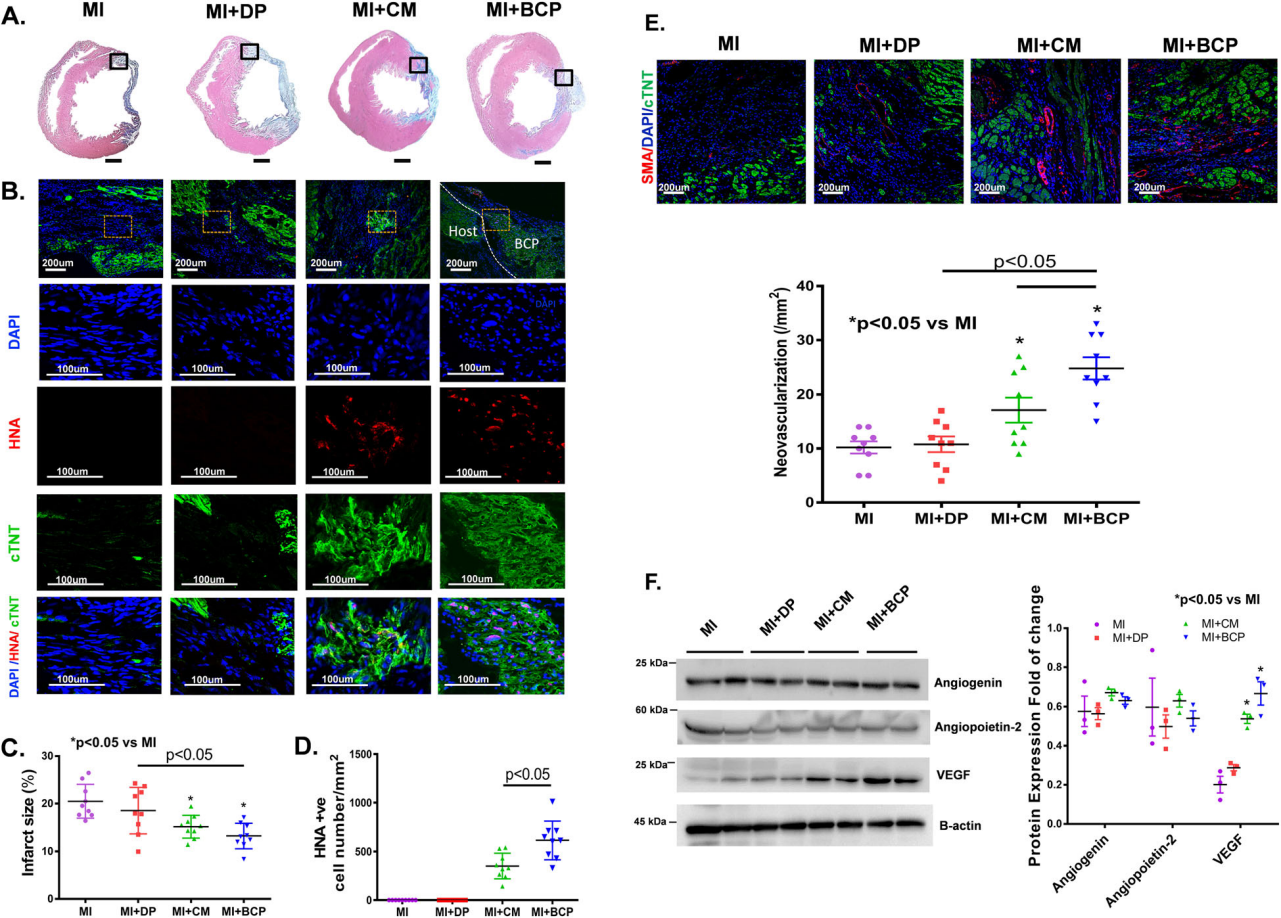
BCP reduced infarct size via improvement of hiPSC-CM survival after transplantation. **a** Representative Masson’s trichrome staining of heart sections 4 weeks after DP, hiPSC-CM, and BCP transplantation. **b** Representative immunofluorescent staining of human nuclei antigen (HNA; red), cardiac troponin T (cTnT; green), and DAPI (blue) at the peri-infarct zone after DP, hiPSC-CM, and BCP transplantation. **c** Infarct size as measured by the percentage area with collagen deposition by trichrome staining indicating that hiPSC-CMs and BCP reduced the infarct size 4 weeks after of transplantation. **d** The quantitative data of HNA and cTNT-positive cells indicate that hiPSC-CMs in the BCP had an improved survival rate compared with hiPSC-CMs only; Student’s *t* test was used to compare MI + CM and MI + BCP groups (*n* = 9; *P <* 0.05). **e** Co-immunofluorescent staining of a-smooth muscle actin (a-SMA) and cTnT in the peri-infarct zone from after MI alone and with DP, hiPSC-CM, and BCP transplantation showed increased neovascularization following BCP transplantation. Data are shown as mean ± SEM. *n* = 9, Four-group comparisons were performed using one-way ANOVA followed by Tukey’s post hoc test. *P < 0.05 compared with MI group. **f** Representative immunoblot (left panel) and protein expression (right panel) of angiogenin, angiopoitin-2, and VEGF to demonstrate that VEGF, but not angiogenin or angiopoietin-2, were significantly increased in the peri-infarct area after transplantation of hiPSC-CMs and BCP compared with the MI group. Four-group comparisons were performed using one-way ANOVA followed by Tukey’s post hoc test (*n* = 3; *P < 0.05 compare to MI group)

Histological examination revealed that transplantation of hiPSC-CMs or a BCP, but not DP, significantly reduced infarct size compared with the MI group (Fig. 5a, c, *P <* 0.05). Immunohistochemical staining demonstrated successful engraftment of hiPSC-CMs at the peri-infarct site after transplantation of hiPSC-CMs or BCP (Fig. 5b). Compared with direct hiPSC-CM transplantation, a BCP significantly improved the engraftment of hiPSC-CMs (Fig. 5d, *P <* 0.05).

To determine whether the improved neovascularization could contribute to the functional improvements after BCP transplantation, α-smooth muscle actin staining was performed at the infarct and peri-infarct area after transplantation. Compared with the MI and MI + DP groups, the vessel density was significantly higher in the peri-infarct area in the MI + CM and MI + BCP groups. Moreover, transplantation of a BCP further increased vessel density compared with transplantation of DP or hiPSC-CMs alone (Fig. 5e, *P <* 0.05). Western blotting was used to determine whether angiogenic growth factors upregulated in the BCP in vivo were also expressed at the infarct and peri-infarct area after transplantation. As shown in Fig. 5f, only the protein level of VEGF, not angiogenin or angiopoietin-2, were significantly increased in the infarct and peri-infarct area after transplantation of hiPSC-CMs and BCP compared with the MI group.

These findings demonstrate that transplantation of a BCP is superior to DP or hiPSC-CMs alone for reducing infarct size and improving neovascularization in post-MI heart failure.

## Discussion

In this study, we have demonstrated that DP can be used as a natural scaffold with hiPSC-CMs to generate a BCP for treatment of post-MI heart failure. First, our results showed that the ECM of DP contained many important growth and pro-angiogenic factors to enhance the survival and maturation of transplanted hiPSC-CMs and promote their paracrine functions. Second, in vitro electrophysiological studies demonstrated that a BCP had synchronized electromechanical properties with electrical stimulation. Third, transplantation of a BCP in an animal model of MI improved the cellular engraftment of hiPSC-CMs, as well as their paracrine effects on neovascularization compared with direct intramyocardial injection. As a result, BCP was superior to DP or hiPSC-CM transplantation for improvement of LV function after MI.

Post-MI heart failure remains a major cause of mortality and morbidity worldwide [26]. Despite the initial promising results of preclinical and clinical studies [27, 28], stem cell-based therapy for cardiac regeneration is limited by inefficient delivery, engraftment, and differentiation of cells in the myocardium after transplantation. Furthermore, as heart failure progresses, the ECM is also modified and replaced by scar tissue that further decreases the survival of transplanted cells [29]. As a result, tissue engineering using different biomaterials and cell types is being developed as a potential therapeutic approach to enhance the efficiency of stem cell therapy by increasing transplanted cell survival and retention [30].

Although various ECM analogs such as synthetic scaffolds [31] and natural biopolymers [32] have been extensively investigated for tissue engineering applications, they all lack the abundant growth factors, complex biochemical properties, and 3D ultrastructure of native mammalian ECM. Accordingly, the ECM derived from decellularized organs has been explored as a promising material for tissue engineering. The decellularization process aims to remove the existing cells from the ECM and thus remove the potential antigens that can induce an inflammatory response and immune-mediated rejection [33]. Natural decellularized ECM not only provides a physical scaffold to maintain the structural integrity of multicellular organisms, it also serves as a reservoir for biochemical and biophysical signals to support cell survival, migration, and differentiation [34]. The main benefit of a decellularized organ or tissue is the presence of 3D fibrous and porous topographies as well as macrostructures like the vasculature, making them an ideal scaffold material for tissue regeneration. Previous studies also have shown that the explanted heart can be decellularized and used to generate human cardiac patches for tissue engineering [15]. Nonetheless, the application of natural heart ECM is limited by the availability of explanted human hearts.

Human placentas are a potential novel source of stem cells and ECM as they are considered medical waste in hospitals and birthing centers. Indeed, placentas have been used as a source of different stem cells such as mesenchymal stem cells [35]. In addition to abundant ECM components, the placenta contains multiple endogenous growth factors, thus DP is a potential novel biomaterial for tissue regeneration [35]. Placenta-derived ECM sheets have been shown to provide a microenvironment that is favorable to the growth and differentiation of stem cells [34]. Intramyocardial injection of placenta-derived ECM into an animal model of MI has been shown to reduce infarct size and attenuate post-MI LV remodeling [20]. Nevertheless, it remains unclear whether the DP can act as a scaffold to improve the survival, maturation, and function of transplanted stem cells to create a BCP for cardiac regeneration.

In this study, a custom-made perfusion system was established to achieve placenta decellularization with minimal disruption to the intrinsic architecture of placenta. Histological examination after placenta decellularization showed that the majority of the cellular components were removed while retaining the structural integrity and composition of the scaffold. The loose and porous property of DP provides a suitable environment for cell attachment and growth. Indeed, we have successfully seeded hiPSC-CMs onto the DP to create a BCP. More importantly, the ECM of DP retained multiple growth and pro-angiogenic factors, even after decellularization, to improve the survival and maturation of transplanted hiPSC-CMs. Comprehensive RNA sequencing revealed that multiple cardiomyocyte structural-related gene, cardiac conduction-related gene, cellular metabolism-related gene, and cell maturation-related gene were upregulated in the BCP compared with hiPSC-CMs alone. Morphological analysis also confirmed that hiPSC-CMs in the BCP exhibited a more mature cardiomyocyte phenotype. Optical mapping also demonstrated that a BCP had spontaneous contraction as well as synchronized electromechanical properties when electrically stimulated.

Despite a similar reduction in infarct size with BCP and hiPSC-CM transplantation, our results demonstrated that BCP was superior to hiPSC-CMs in terms of improved LV function following MI. It is likely the improvement in cellular maturation and engraftment of hiPSC-CMs into the infarct site will lead to increased LV contractile function. In addition, the transplantation of BCP was superior to hiPSC-CMs alone in enhancement of neovascularization at the peri-infarct regions via its paracrine mechanisms with increased expression of VEGF. Indeed, our in vitro analysis also confirmed that the paracrine function of hiPSC-CMs in expression of angiogenic growth factors was significantly improved after hiPSC-CMs seeding onto DP scaffold to generate a BCP.

This study has several limitations. First, previous studies have shown that other methods such as mechanical stretching can improve the maturation and function of a BCP [36]. It is unclear whether further manipulation of our BCP using DP further improves its therapeutic efficacy. Second, the potential arrhythmic risk of a BCP is unknown since the rapid heart rate of mice will mask the risk of ventricular arrhythmias of hiPSC-CMs with a lower resting heart rate after transplantation. Therefore, our results need to be confirmed in a large animal model of MI, e.g., swine or non-human primate, with a similar heart rate to humans. Third, the various soluble factors secreted from BCP and monolayer cultured hiPSC-CMs may bring therapy benefit to the injured myocardium and have not been fully investigated in present study, their potential value in the myocardial repair worth to further evaluate and compare with the hiPSC-CMs and BCP transplantation. We will evaluate the application of those acellular soluble factors in myocardial repair via future study.

## Conclusions

In summary, our results provide important proof-of-principle data to support the use of DP as a scaffold material to generate a BCP using hiPSC-CMs to improve their survival, maturation, engraftment, and paracrine functions for cardiac regeneration following MI.

## Supplementary Information

**Additional file 1: Supplementary Table S1.** PCR primer sequences.

**Additional file 2: Supplementary Figure S1.** BCP was surgically sutured onto the surface of the epicardium on day1 and engrafted into the myocardium on day 28.

**Additional file 3: Supplementary Figure S2.** A. Representative M-Mode echocardiographic images; and B. Pressure-volume loop tracing in control, MI, MI + DP, MI + CM and BCP groups 4 weeks after transplantation.

**Additional file 4: Supplementary Figure S3.** A. After 7 days of cell seeding, BCP was fixed and stained with cTnT, DAPI and collagen I, images were obtained using a confocal microscope. B. DNA content analysis to evaluate the efficiency of decellularization. Total DNA of native rat placenta, DP and BCP was extracted and compared, quantitatively data indicate the DNA content was removed from DP and regenerated after cell seeding. Error bars represent mean SEM of Five independent experiments. Three-group comparisons were performed using one-way ANOVA followed by the Tukey’s post hoc test. *, *p < 0.05* compare with native placenta.

**Additional file 5: Supplementary Figure S4.** A. RNA sequencing was performed to evaluate the gene expression difference in monolayer cultured hiPSC-CMs and BCP. B. The number of up (3136) or down (2543) regulated genes depicted in monolayer cultured hiPSC-CMs compared with BCP.

## Data Availability

The data that support the findings of this study are available from the corresponding author upon reasonable request.

## Abbreviations

BCP: Bioengineered cardiac patches
cTnT: Cardiac troponin T
DP: Decellularized placenta
ECM: Extracellular matrix
ESPVR: End-systolic pressure-volume relationship
FS: Fractional shortening
hiPSC: Human-induced pluripotent stem cell
hiPSC-CMs: Human-induced pluripotent stem cell-derived cardiomyocytes
LV: Left ventricular
LVEF: Left ventricular ejection fraction
LVEDD: Left ventricular end-diastolic diameter
LVESD: Left ventricular end-systolic diameter
LVEDP: Left ventricular end-diastolic pressure
LVESP: Left ventricular end-systolic pressure
MI: Myocardial infarction
VEGF: Vascular endothelial growth factor
+ dP/dt _max_: Maximal positive derivatives of left ventricular pressure
