# Supplementary figures and images for: Myocardial repair of bioengineered cardiac patches with decellularized placental scaffold and human-induced pluripotent stem cells in a rat model of myocardial infarction
Source: Stem Cell Res Ther. 2021 Jan 7;12:13. doi: 10.1186/s13287-020-02066-y (PMC7791702; doi:10.1186/s13287-020-02066-y)

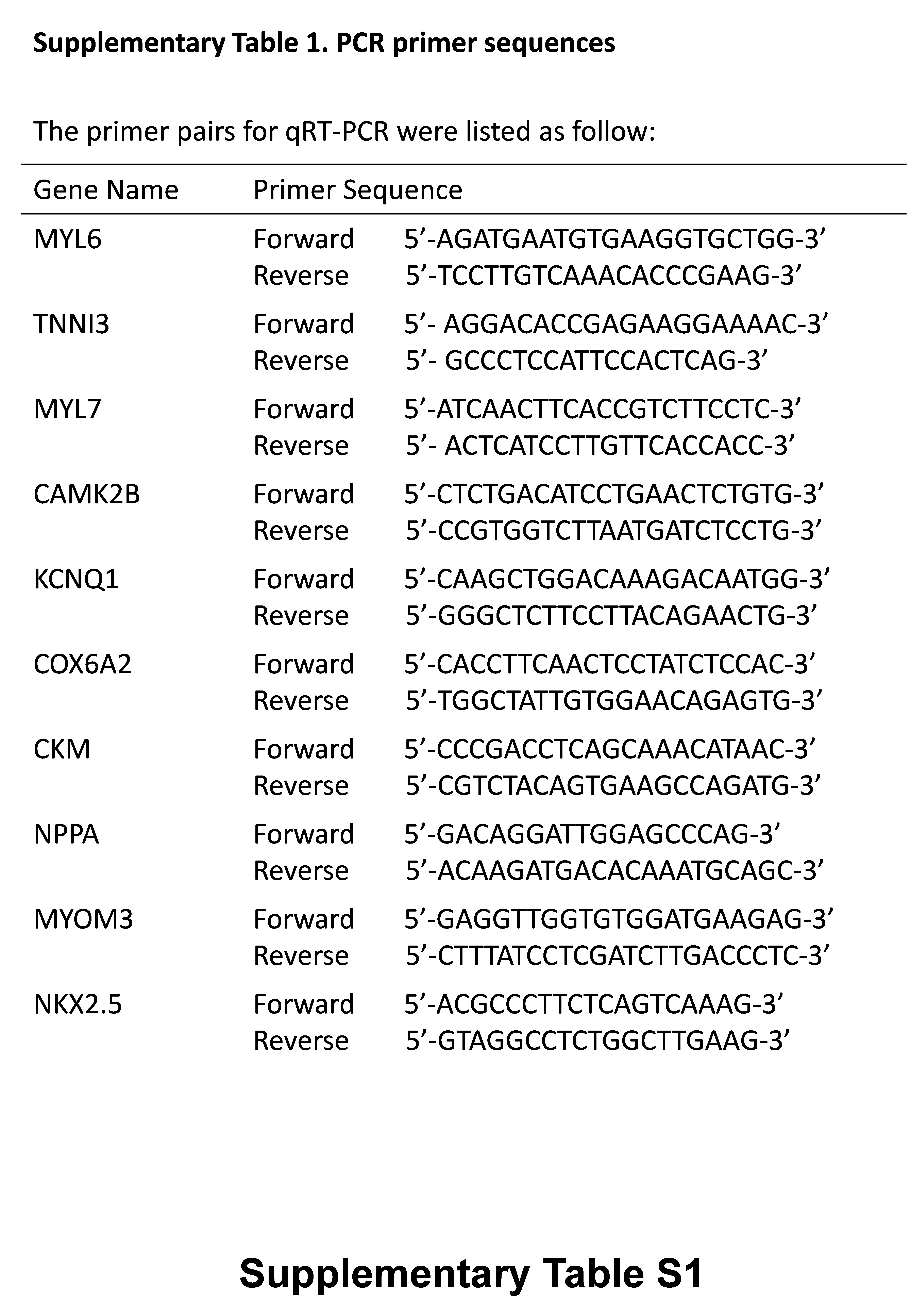

Supplement: Supplementary file 4 — Additional file 1: Supplementary Table S1. PCR primer sequences. [file 13287_2020_2066_MOESM1_ESM.jpg]

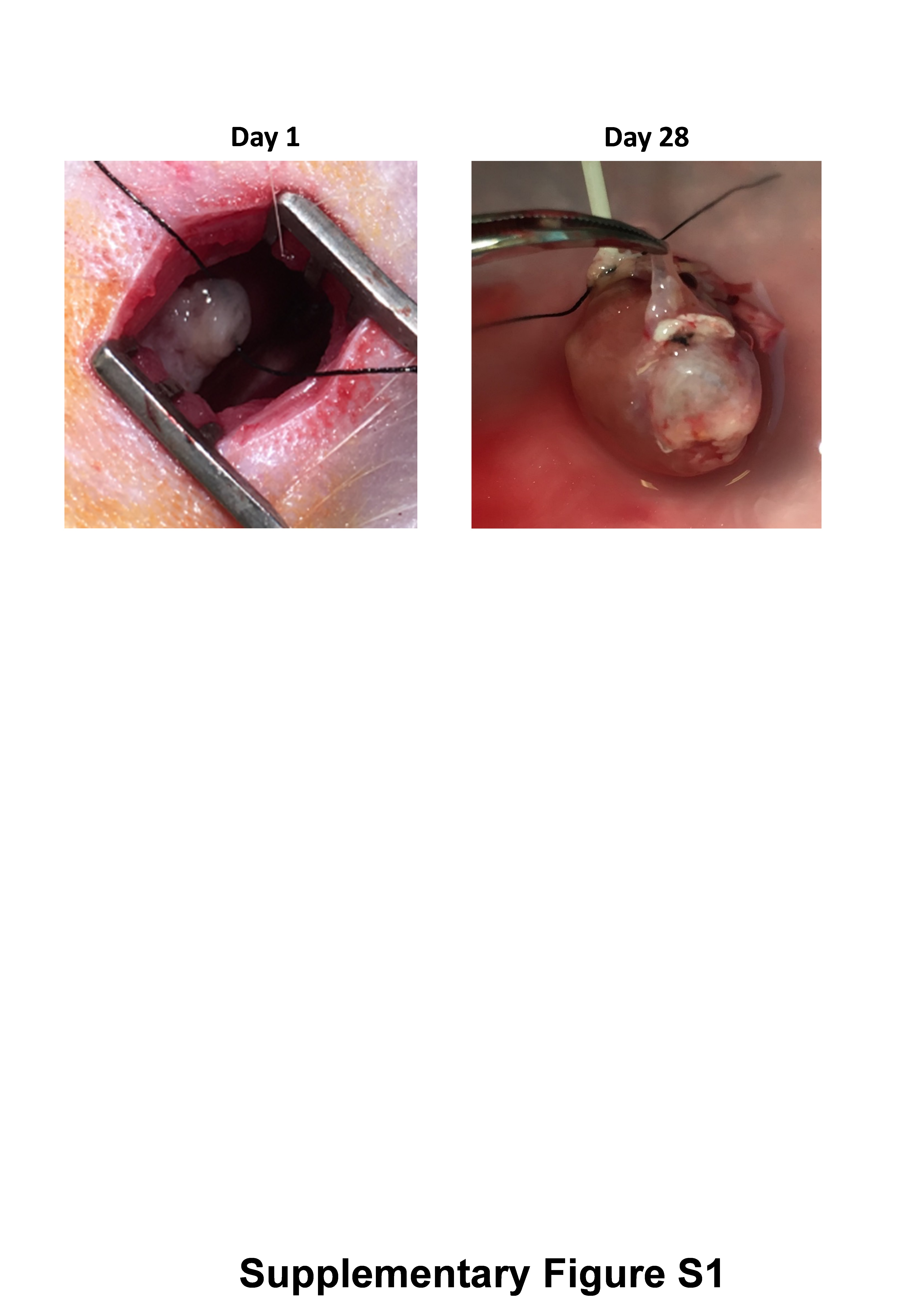

Supplement: Supplementary file 5 — Additional file 2: Supplementary Figure S1. BCP was surgically sutured onto the surface of the epicardium on day1 and engrafted into the myocardium on day 28. [file 13287_2020_2066_MOESM2_ESM.jpg]

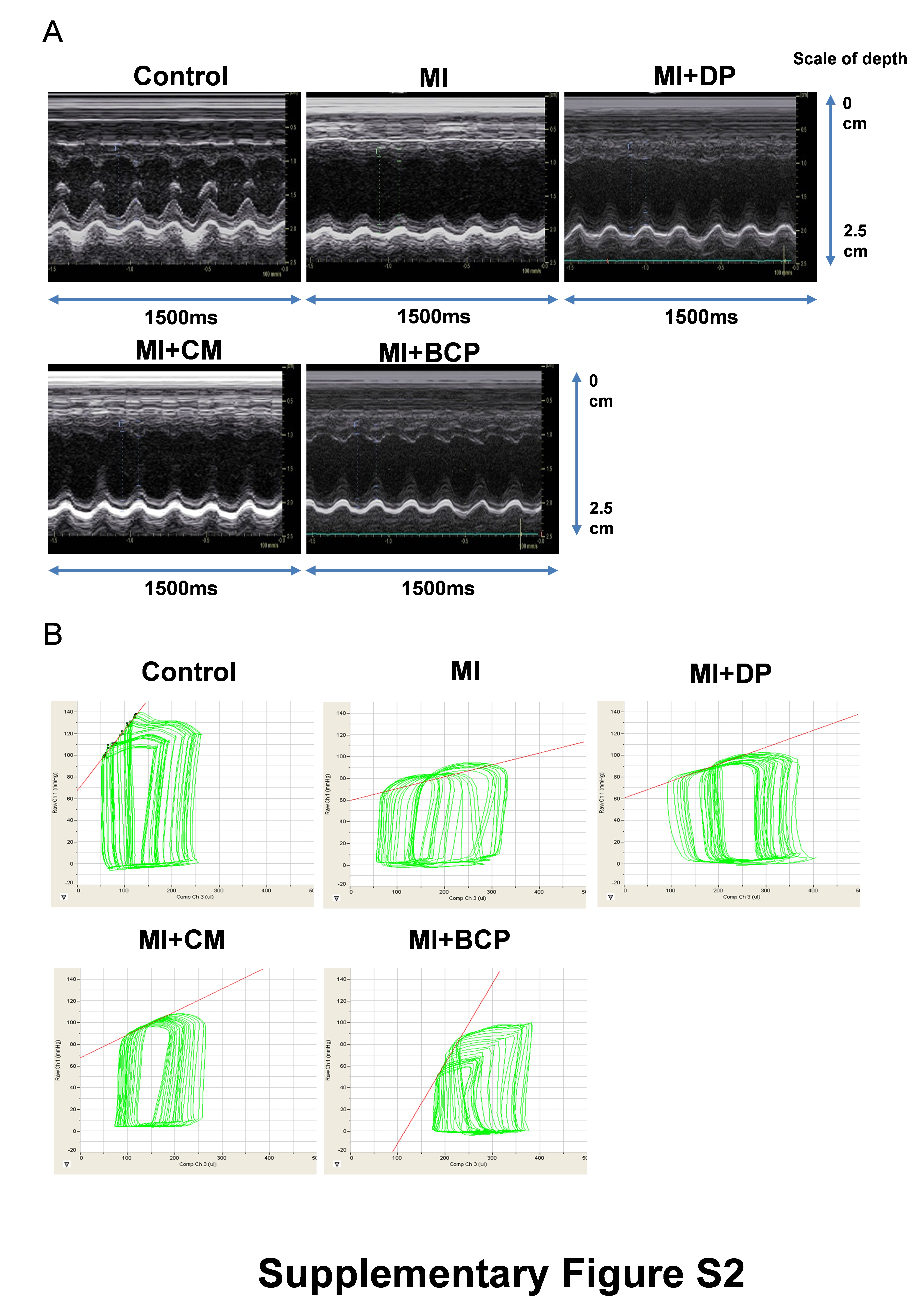

Supplement: Supplementary file 6 — Additional file 3: Supplementary Figure S2. A. Representative M-Mode echocardiographic images; and B. Pressure-volume loop tracing in control, MI, MI + DP, MI + CM and BCP groups 4 weeks after transplantation. [file 13287_2020_2066_MOESM3_ESM.jpg]

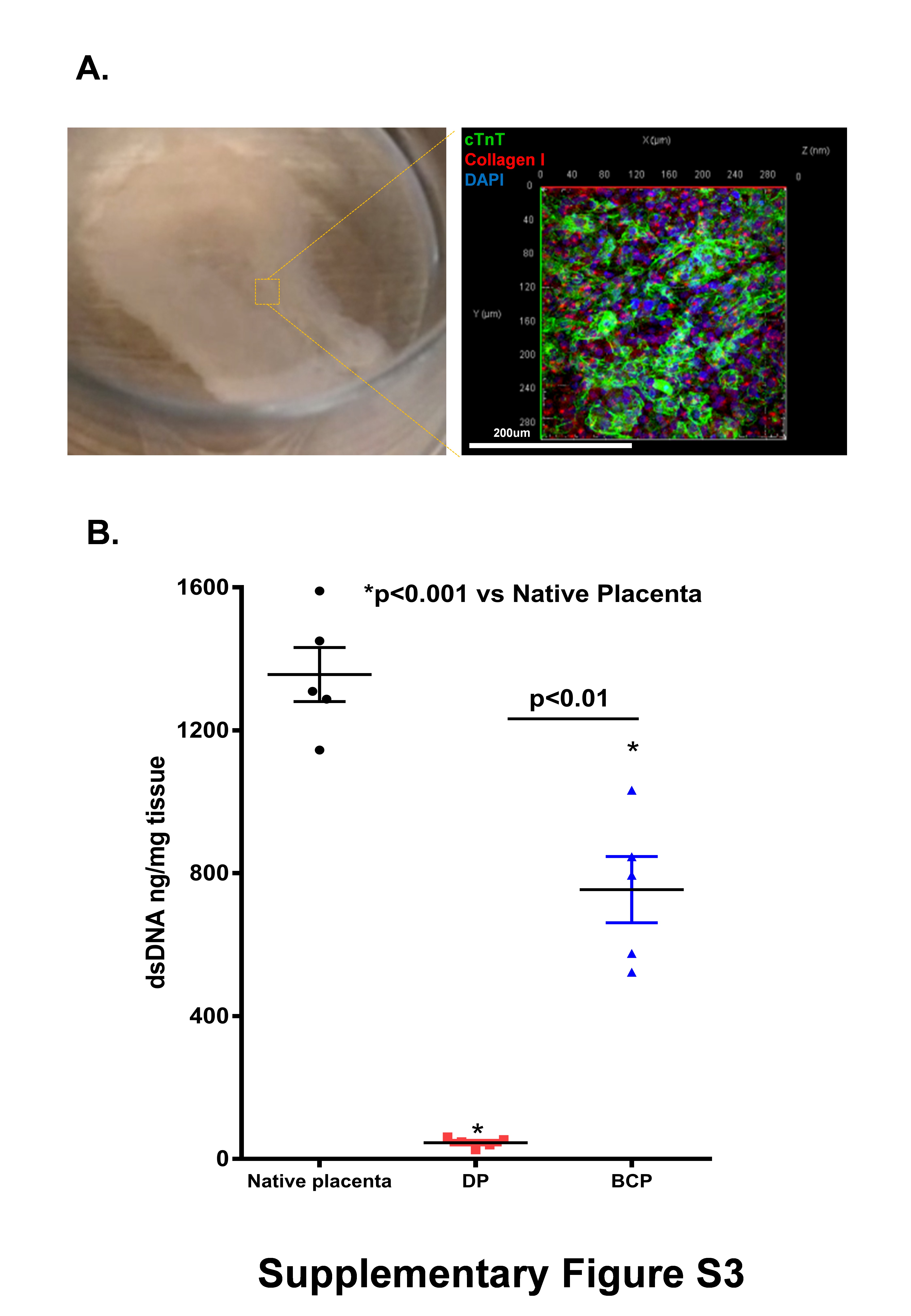

Supplement: Supplementary file 7 — Additional file 4: Supplementary Figure S3. A. After 7 days of cell seeding, BCP was fixed and stained with cTnT, DAPI and collagen I, images were obtained using a confocal microscope. B. DNA content analysis to evaluate the efficiency of decellularization. Total DNA of native rat placenta, DP and BCP was extracted and compared, quantitatively data indicate the DNA content was removed from DP and regenerated after cell seeding. Error bars represent mean SEM of Five independent experiments. Three-group comparisons were performed using one-way ANOVA followed by the Tukey’s post hoc test. *, p < 0.05 compare with native placenta. [file 13287_2020_2066_MOESM4_ESM.jpg]

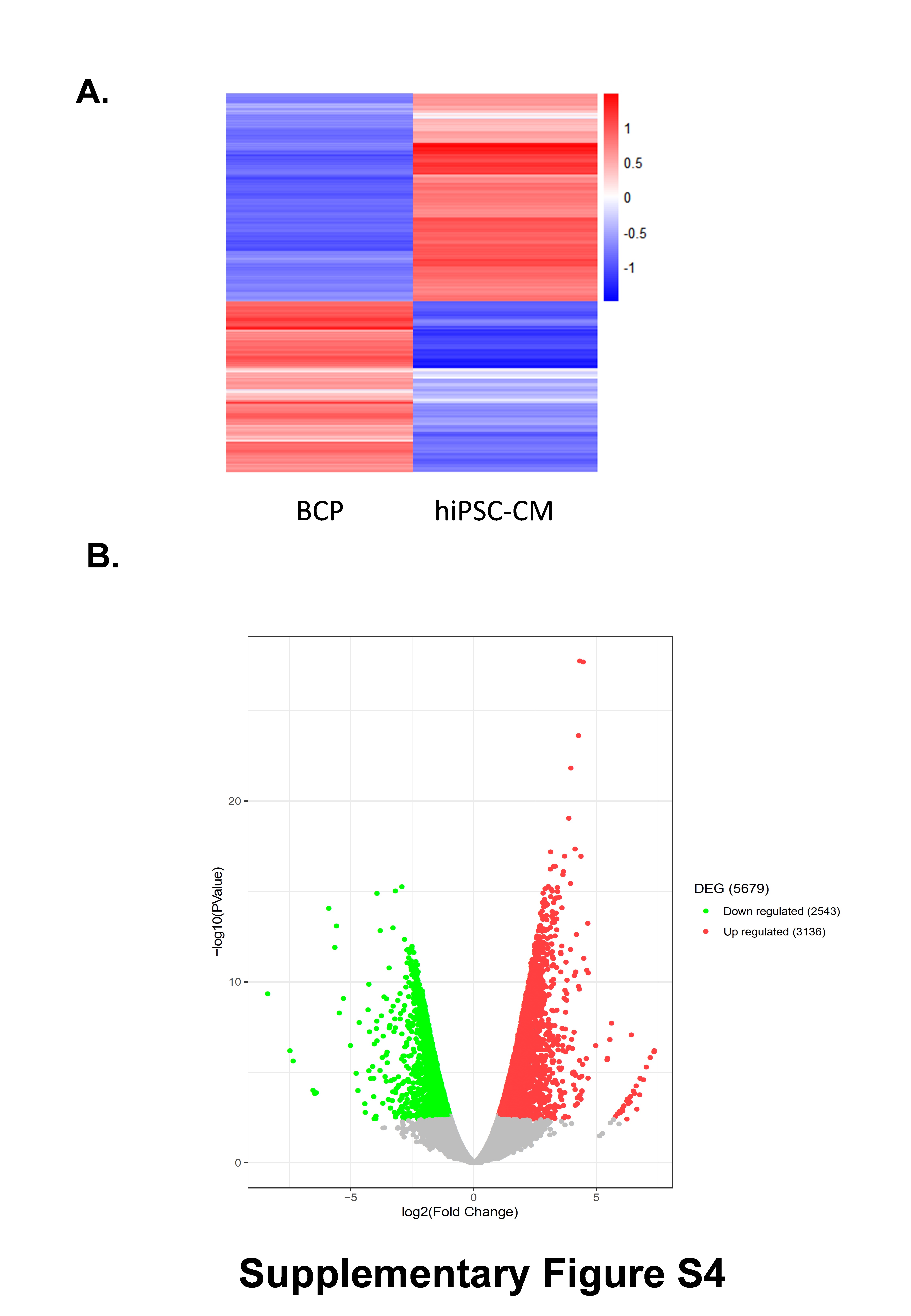

Supplement: Supplementary file 8 — Additional file 5: Supplementary Figure S4. A. RNA sequencing was performed to evaluate the gene expression difference in monolayer cultured hiPSC-CMs and BCP. B. The number of up (3136) or down (2543) regulated genes depicted in monolayer cultured hiPSC-CMs compared with BCP. [file 13287_2020_2066_MOESM5_ESM.jpg]
